# Machine learning applications in cancer prognosis and prediction

**DOI:** 10.1016/j.csbj.2014.11.005

**Published:** 2014-11-15

**Authors:** Konstantina Kourou, Themis P. Exarchos, Konstantinos P. Exarchos, Michalis V. Karamouzis, Dimitrios I. Fotiadis

**Affiliations:** a Unit of Medical Technology and Intelligent Information Systems, Dept. of Materials Science and Engineering, University of Ioannina, Ioannina, Greece; b IMBB — FORTH, Dept. of Biomedical Research, Ioannina, Greece; c Molecular Oncology Unit, Department of Biological Chemistry, Medical School, University of Athens, Athens, Greece

**Keywords:** ML, Machine Learning, ANN, Artificial Neural Network, SVM, Support Vector Machine, DT, Decision Tree, BN, Bayesian Network, SSL, Semi-supervised Learning, TCGA, The Cancer Genome Atlas Research Network, HTT, High-throughput Technologies, OSCC, Oral Squamous Cell Carcinoma, CFS, Correlation based Feature Selection, AUC, Area Under Curve, ROC, Receiver Operating Characteristic, BCRSVM, Breast Cancer Support Vector Machine, PPI, Protein–Protein Interaction, GEO, Gene Expression Omnibus, LCS, Learning Classifying Systems, ES, Early Stopping algorithm, SEER, Surveillance, Epidemiology and End results Database, NSCLC, Non-small Cell Lung Cancer, NCI caArray, National Cancer Institute Array Data Management System, Machine learning, Cancer susceptibility, Predictive models, Cancer recurrence, Cancer survival

## Abstract

Cancer has been characterized as a heterogeneous disease consisting of many different subtypes. The early diagnosis and prognosis of a cancer type have become a necessity in cancer research, as it can facilitate the subsequent clinical management of patients. The importance of classifying cancer patients into high or low risk groups has led many research teams, from the biomedical and the bioinformatics field, to study the application of machine learning (ML) methods. Therefore, these techniques have been utilized as an aim to model the progression and treatment of cancerous conditions. In addition, the ability of ML tools to detect key features from complex datasets reveals their importance. A variety of these techniques, including Artificial Neural Networks (ANNs), Bayesian Networks (BNs), Support Vector Machines (SVMs) and Decision Trees (DTs) have been widely applied in cancer research for the development of predictive models, resulting in effective and accurate decision making. Even though it is evident that the use of ML methods can improve our understanding of cancer progression, an appropriate level of validation is needed in order for these methods to be considered in the everyday clinical practice. In this work, we present a review of recent ML approaches employed in the modeling of cancer progression. The predictive models discussed here are based on various supervised ML techniques as well as on different input features and data samples. Given the growing trend on the application of ML methods in cancer research, we present here the most recent publications that employ these techniques as an aim to model cancer risk or patient outcomes.

## 1 Introduction

Over the past decades, a continuous evolution related to cancer research has been performed [1]. Scientists applied different methods, such as screening in early stage, in order to find types of cancer before they cause symptoms. Moreover, they have developed new strategies for the early prediction of cancer treatment outcome. With the advent of new technologies in the field of medicine, large amounts of cancer data have been collected and are available to the medical research community. However, the accurate prediction of a disease outcome is one of the most interesting and challenging tasks for physicians. As a result, ML methods have become a popular tool for medical researchers. These techniques can discover and identify patterns and relationships between them, from complex datasets, while they are able to effectively predict future outcomes of a cancer type.

Given the significance of personalized medicine and the growing trend on the application of ML techniques, we here present a review of studies that make use of these methods regarding the cancer prediction and prognosis. In these studies prognostic and predictive features are considered which may be independent of a certain treatment or are integrated in order to guide therapy for cancer patients, respectively [2]. In addition, we discuss the types of ML methods being used, the types of data they integrate, the overall performance of each proposed scheme while we also discuss their pros and cons.

An obvious trend in the proposed works includes the integration of mixed data, such as clinical and genomic. However, a common problem that we noticed in several works is the lack of external validation or testing regarding the predictive performance of their models. It is clear that the application of ML methods could improve the accuracy of cancer susceptibility, recurrence and survival prediction. Based on [3], the accuracy of cancer prediction outcome has significantly improved by 15%–20% the last years, with the application of ML techniques.

Several studies have been reported in the literature and are based on different strategies that could enable the early cancer diagnosis and prognosis [4], [5], [6], [7]. Specifically, these studies describe approaches related to the profiling of circulating miRNAs that have been proven a promising class for cancer detection and identification. However, these methods suffer from low sensitivity regarding their use in screening at early stages and their difficulty to discriminate benign from malignant tumors. Various aspects regarding the prediction of cancer outcome based on gene expression signatures are discussed in [8], [9]. These studies list the potential as well as the limitations of microarrays for the prediction of cancer outcome. Even though gene signatures could significantly improve our ability for prognosis in cancer patients, poor progress has been made for their application in the clinics. However, before gene expression profiling can be used in clinical practice, studies with larger data samples and more adequate validation are needed.

In the present work only studies that employed ML techniques for modeling cancer diagnosis and prognosis are presented.

## 2 ML techniques

ML, a branch of Artificial Intelligence, relates the problem of learning from data samples to the general concept of inference [10], [11], [12]. Every learning process consists of two phases: (i) estimation of unknown dependencies in a system from a given dataset and (ii) use of estimated dependencies to predict new outputs of the system. ML has also been proven an interesting area in biomedical research with many applications, where an acceptable generalization is obtained by searching through an *n*-*dimensional* space for a given set of biological samples, using different techniques and algorithms [13]. There are two main common types of ML methods known as (i) supervised learning and (ii) unsupervised learning. In supervised learning a labeled set of training data is used to estimate or map the input data to the desired output. In contrast, under the unsupervised learning methods no labeled examples are provided and there is no notion of the output during the learning process. As a result, it is up to the learning scheme/model to find patterns or discover the groups of the input data. In supervised learning this procedure can be thought as a classification problem. The task of classification refers to a learning process that categorizes the data into a set of finite classes. Two other common ML tasks are regression and clustering. In the case of regression problems, a learning function maps the data into a real-value variable. Subsequently, for each new sample the value of a predictive variable can be estimated, based on this process. Clustering is a common unsupervised task in which one tries to find the categories or clusters in order to describe the data items. Based on this process each new sample can be assigned to one of the identified clusters concerning the similar characteristics that they share.

**Fig. 1:**
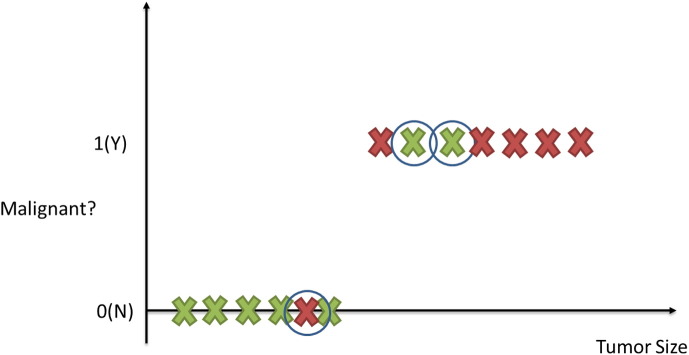
Classification task in supervised learning. Tumors are represented as X and classified as benign or malignant. The circled examples depict those tumors that have been misclassified.

Suppose for example that we have collected medical records relevant to breast cancer and we try to predict if a tumor is malignant or benign based on its size. The ML question would be referred to the estimation of the probability that the tumor is malignant or no (1 = Yes, 0 = No). Fig. 1 depicts the classification process of a tumor being malignant or not. The circled records depict any misclassification of the type of a tumor produced by the procedure.

Another type of ML methods that have been widely applied is semi-supervised learning, which is a combination of supervised and unsupervised learning. It combines labeled and unlabeled data in order to construct an accurate learning model. Usually, this type of learning is used when there are more unlabeled datasets than labeled.

When applying a ML method, data samples constitute the basic components. Every sample is described with several features and every feature consists of different types of values. Furthermore, knowing in advance the specific type of data being used allows the right selection of tools and techniques that can be used for their analysis. Some data-related issues refer to the quality of the data and the preprocessing steps to make them more suitable for ML. Data quality issues include the presence of noise, outliers, missing or duplicate data and data that is biased-unrepresentative. When improving the data quality, typically the quality of the resulting analysis is also improved. In addition, in order to make the raw data more suitable for further analysis, preprocessing steps should be applied that focus on the modification of the data. A number of different techniques and strategies exist, relevant to data preprocessing that focus on modifying the data for better fitting in a specific ML method. Among these techniques some of the most important approaches include (i) dimensionality reduction (ii) feature selection and (iii) feature extraction. There are many benefits regarding the dimensionality reduction when the datasets have a large number of features. ML algorithms work better when the dimensionality is lower [14]. Additionally, the reduction of dimensionality can eliminate irrelevant features, reduce noise and can produce more robust learning models due to the involvement of fewer features. In general, the dimensionality reduction by selecting new features which are a subset of the old ones is known as feature selection. Three main approaches exist for feature selection namely embedded, filter and wrapper approaches [14]. In the case of feature extraction, a new set of features can be created from the initial set that captures all the significant information in a dataset. The creation of new sets of features allows for gathering the described benefits of dimensionality reduction.

However, the application of feature selection techniques may result in specific fluctuations concerning the creation of predictive feature lists. Several studies in the literature discuss the phenomenon of lack of agreement between the predictive gene lists discovered by different groups, the need of thousands of samples in order to achieve the desired outcomes, the lack of biological interpretation of predictive signatures and the dangers of information leak recorded in published studies [15], [16], [17], [18].

The main objective of ML techniques is to produce a model which can be used to perform classification, prediction, estimation or any other similar task. The most common task in learning process is classification. As mentioned previously, this learning function classifies the data item into one of several predefined classes. When a classification model is developed, by means of ML techniques, training and generalization errors can be produced. The former refers to misclassification errors on the training data while the latter on the expected errors on testing data. A good classification model should fit the training set well and accurately classify all the instances. If the test error rates of a model begin to increase even though the training error rates decrease then the phenomenon of model overfitting occurs. This situation is related to model complexity meaning that the training errors of a model can be reduced if the model complexity increases. Obviously, the ideal complexity of a model not susceptible to overfitting is the one that produces the lowest generalization error. A formal method for analyzing the expected generalization error of a learning algorithm is the bias–variance decomposition. The bias component of a particular learning algorithm measures the error rate of that algorithm. Additionally, a second source of error over all possible training sets of given size and all possible test sets is called variance of the learning method. The overall expected error of a classification model is constituted of the sum of bias and variance, namely the bias–variance decomposition.

**Fig. 2:**
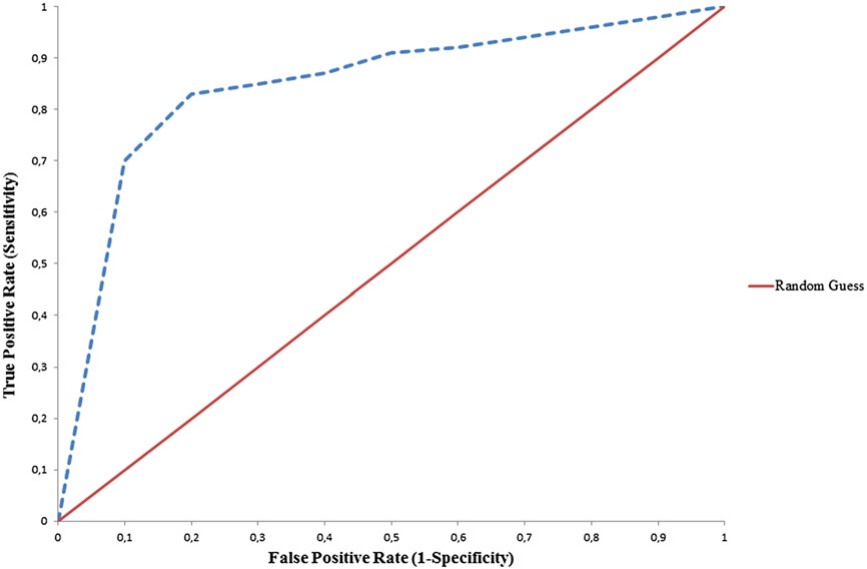
An indicative ROC curve of two classifiers: (a) Random Guess classifier (red curve) and (b) A classifier providing more robust predictions (blue dotted curve).

Once a classification model is obtained using one or more ML techniques, it is important to estimate the classifier's performance. The performance analysis of each proposed model is measured in terms of sensitivity, specificity, accuracy and area under the curve (AUC). Sensitivity is defined as the proportion of true positives that are correctly observed by the classifier, whereas specificity is given by the proportion of true negatives that are correctly identified. The quantitative metrics of accuracy and AUC are used for assessing the overall performance of a classifier. Specifically, accuracy is a measure related to the total number of correct predictions. On the contrary, AUC is a measure of the model's performance which is based on the ROC curve that plots the tradeoffs between sensitivity and 1-specificity (Fig. 2).

The predictive accuracy of the model is computed from the testing set which provides an estimation of the generalization errors. In order to obtain reliable results regarding the predicting performance of a model, training and testing samples should be sufficiently large and independent while the labels of the testing sets should be known. Among the most commonly used methods for evaluating the performance of a classifier by splitting the initial labeled data into subsets are: (i) Holdout Method, (ii) Random Sampling, (iii) Cross-Validation and (iv) Bootstrap. In the Holdout method, the data samples are partitioned into two separate sets, namely the training and the test sets. A classification model is then generated from the training set while its performance is estimated on the test set. Random sampling is a similar approach to the Holdout method. In this case, in order to better estimate the accuracy, the Holdout method is repeated several times, choosing the training and test instances randomly. In the third approach, namely cross-validation, each sample is used the same number of times for training and only once for testing. As a result, the original data set is covered successfully both in the training and in the test set. The accuracy results are calculated as the average of all different validation cycles. In the last approach, bootstrap, the samples are separated with replacement into training and test sets, i.e. they are placed again into the entire data set after they have been chosen for training.

When the data are preprocessed and we have defined the kind of learning task, a list of ML methods including (i) ANNs, (ii) DTs, (iii) SVMs and (iv) BNs is available. Based on the intension of this review paper, we will refer only to these ML techniques that have been applied widely in the literature for the case study of cancer prediction and prognosis. We identify the trends regarding the types of ML methods that are used, the types of data that are integrated as well as the evaluation methods employed for assessing the overall performance of the methods used for cancer prediction or disease outcomes.

**Fig. 3:**
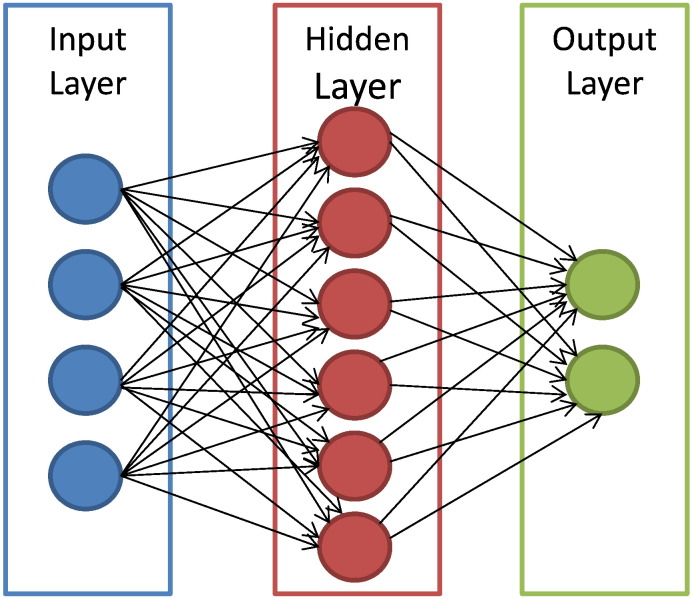
An illustration of the ANN structure. The arrows connect the output of one node to the input of another.

ANNs handle a variety of classification or pattern recognition problems. They are trained to generate an output as a combination between the input variables. Multiple hidden layers that represent the neural connections mathematically are typically used for this process. Even though ANNs serve as a gold standard method in several classification tasks [19] they suffer from certain drawbacks. Their generic layered structure proves to be time-consuming while it can lead to very poor performance. Additionally, this specific technique is characterized as a “black-box” technology. Trying to find out how it performs the classification process or why an ANN did not work is almost impossible to detect. Fig. 3 depicts the structure of an ANN with its interconnected group of nodes.

**Fig. 4:**
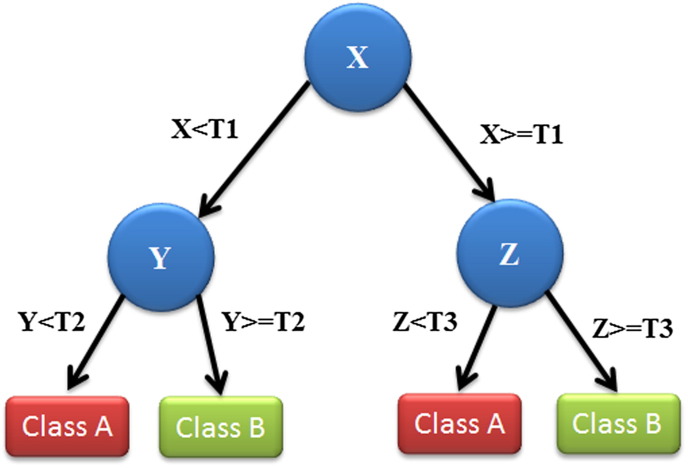
An illustration of a DT showing the tree structure. Each variable (X, Y, Z) is represented by a circle and the decision outcomes by squares (Class A, Class B). T(1–3) represents the thresholds (classification rules) in order to successfully classify each variable to a class label.

DTs follow a tree-structured classification scheme where the nodes represent the input variables and the leaves correspond to decision outcomes. DTs are one of the earliest and most prominent ML methods that have been widely applied for classification purposes. Based on the architecture of the DTs, they are simple to interpret and “quick” to learn. When traversing the tree for the classification of a new sample we are able to conjecture about its class. The decisions resulted from their specific architecture allow for adequate reasoning which makes them an appealing technique. Fig. 4 depicts an illustration of a DT with its elements and rules.

**Fig. 5:**
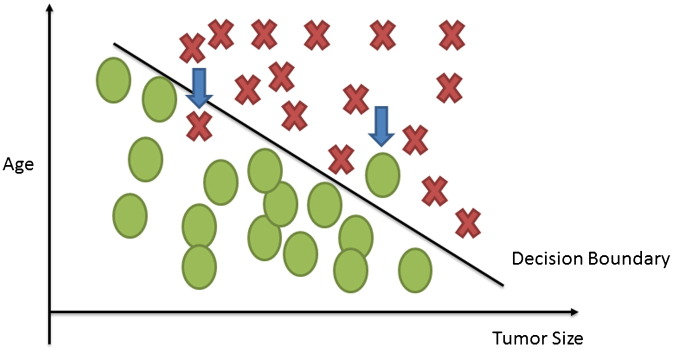
A simplified illustration of a linear SVM classification of the input data. Figure was reproduced from the ML lectures of [21]. Tumors are classified according to their size and the patient's age. The depicted arrows display the misclassified tumors.

SVMs are a more recent approach of ML methods applied in the field of cancer prediction/prognosis. Initially SVMs map the input vector into a feature space of higher dimensionality and identify the hyperplane that separates the data points into two classes. The marginal distance between the decision hyperplane and the instances that are closest to boundary is maximized. The resulting classifier achieves considerable generalizability and can therefore be used for the reliable classification of new samples. It is worth noting that probabilistic outputs can also be obtained for SVMs [20]. Fig. 5 illustrates how an SVM might work in order to classify tumors among benign and malignant based on their size and patients' age. The identified hyperplane can be thought as a decision boundary between the two clusters. Obviously, the existence of a decision boundary allows for the detection of any misclassification produced by the method.

BN classifiers produce probability estimations rather than predictions. As their name reveals, they are used to represent knowledge coupled with probabilistic dependencies among the variables of interest via a directed acyclic graph. BNs have been applied widely to several classification tasks as well as for knowledge representation and reasoning purposes.

**Fig. 6:**
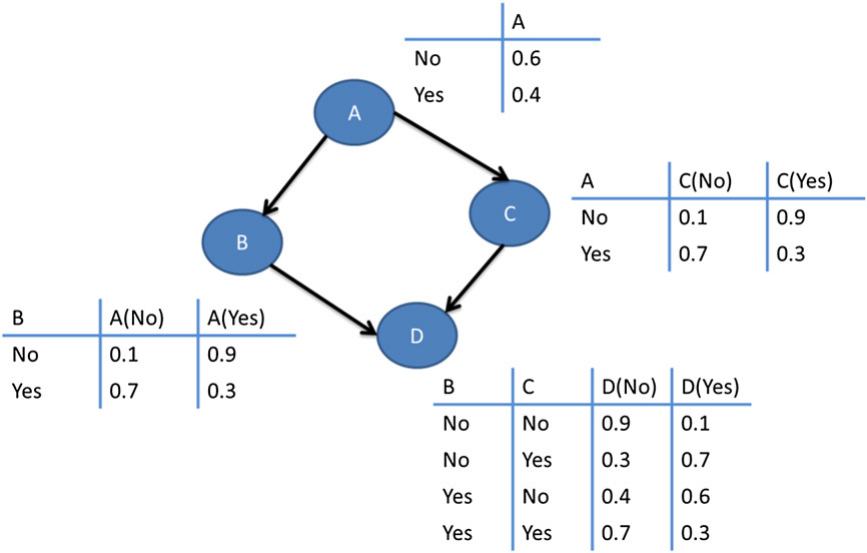
An illustration of a BN. Nodes (A–D) represent a set of random variables across with their conditional probabilities which are calculated in each table.

Fig. 6 depicts an illustration of a BN across with the calculated conditional probability for each variable.

## 3 ML and cancer prediction/prognosis

**Fig. 7:**
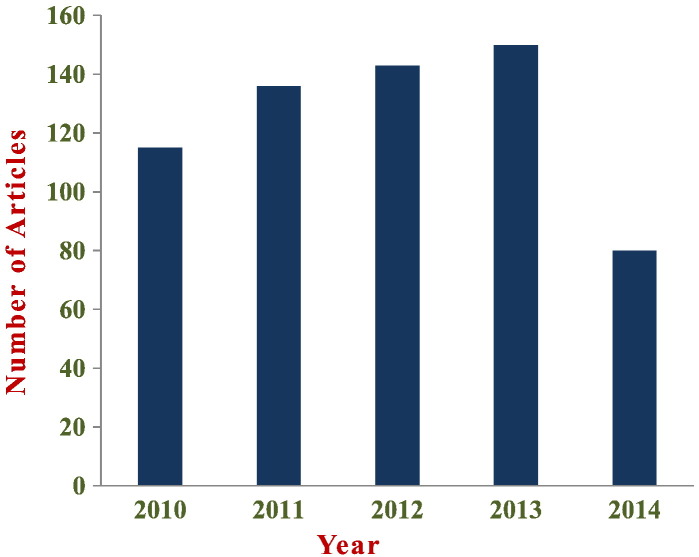
Distribution of published studies, within the last 5 years, that employ ML techniques for cancer prediction.

The last two decades a variety of different ML techniques and feature selection algorithms have been widely applied to disease prognosis and prediction [3], [22], [23], [24], [25], [26], [27]. Most of these works employ ML methods for modeling the progression of cancer and identify informative factors that are utilized afterwards in a classification scheme. Furthermore, in almost all the studies gene expression profiles, clinical variables as well as histological parameters are encompassed in a complementary manner in order to be fed as input to the prognostic procedure. Fig. 7 depicts the distribution in published papers using ML techniques to predict (i) cancer susceptibility, (ii) recurrence and (iii) survival. The information was collected based on a variety of query searches in the Scopus biomedical database. More specifically, queries like “cancer risk assessment” AND “Machine Learning”, “cancer recurrence” AND “Machine Learning”, “cancer survival” AND “Machine Learning” as well as “cancer prediction” AND “Machine Learning” yielded the number of papers that are depicted in Fig. 3. No limitations were imposed in the resulted hits except the exclusion of articles published before 2010. As mentioned above, the number of papers presented in Fig. 7 refers to the exact numbers yielded from the databases without any refinement except the date that they were published.

The success of a disease prognosis is undoubtedly dependent on the quality of a medical diagnosis; however, a prognostic prediction should take into account more than a simple diagnostic decision. When dealing with cancer prognosis/prediction one is concerned with three predictive tasks: (i) the prediction of cancer susceptibility (risk assessment), (ii) the prediction of cancer recurrence/local control and (iii) the prediction of cancer survival. In the first two cases one is trying to find (i) the likelihood of developing a type of cancer and (ii) the likelihood of redeveloping a type of cancer after complete or partial remission. In the last case, the prediction of a survival outcome such as disease-specific or overall survival after cancer diagnosis or treatment is the main objective. The prediction of cancer outcome usually refers to the cases of (i) life expectancy, (ii) survivability, (iii) progression and (iv) treatment sensitivity [3].

Major types of ML techniques including ANNs and DTs have been used for nearly three decades in cancer detection [22], [28], [29], [30]. According to the recent PubMed results regarding the subject of ML and cancer more than 7510 articles have been published until today. The vast majority of these publications makes use of one or more ML algorithms and integrates data from heterogeneous sources for the detection of tumors as well as for the prediction/prognosis of a cancer type. A growing trend is noted the last decade in the use of other supervised learning techniques, namely SVMs and BNs, towards cancer prediction and prognosis [24], [31], [32], [33], [34], [35], [36]. All of these classification algorithms have been widely used in a wide range of problems posed in cancer research.

In the past, the typical information used by the physicians conclude with a reasonable decision regarding cancer prognosis and included histological, clinical and population-based data [23], [37]. The integration of features such as family history, age, diet, weight, high-risk habits and exposure to environmental carcinogens play a critical role in predicting the development of cancer [38], [39], [40]. Even though this type of macro-scale information referred to a small number of variables so that standard statistical methods could be used for prediction purposes, however these types of parameters do not provide sufficient information for making robust decisions. With the rapid advent of genomic, proteomic and imaging technologies a new kind of molecular information can be obtained. Molecular biomarkers, cellular parameters as well as the expression of certain genes have been proven as very informative indicators for cancer prediction. The presence of such High Throughput Technologies (HTTs) nowadays has produced huge amounts of cancer data that are collected and are available to the medical research community. However, the accurate prediction of a disease outcome is one of the most interesting and challenging tasks for physicians. As a result, ML methods have become a popular tool for medical researchers. These techniques can discover and identify patterns and relationships between them, from complex datasets, while they are able to effectively predict future outcomes of a cancer type. Additionally, feature selection methods have been published in the literature with their application in cancer [41], [42], [43]. The proposed computational tools aim at identifying informative features for accurately identification of disease class.

There are nowadays separate subgroups among the same type of cancer based on specific genetic defects that have different treatment approaches and options as well as different clinical outcomes. This is the foundation of the individualized treatment approach, in which computational techniques could help by identifying less costly and effectively such small groups of patients. Furthermore, the development of a community resource project, namely The Cancer Genome Atlas Research Network (TCGA) has the potential support for personal medicine as it provides large scale genomic data about specific tumor types. TCGA provides with the ability to better understand the molecular basis of cancer through the application of high-throughput genome technologies.

## 4 Survey of ML applications in cancer

An extensive search was conducted relevant to the use of ML techniques in cancer susceptibility, recurrence and survivability prediction. Two electronic databases were accessed namely PubMed, Scopus. Due to the vast number of articles returned by the search queries, further scrutinization was needed in order to maintain the most relevant articles. The relevance of each publication was assessed based on the keywords of the three predictive tasks found in their titles and abstracts. Specifically, after reading their titles and abstracts we only selected those publications that study one of the three foci of cancer prediction and included it in their titles. The majority of these studies use different types of input data: genomic, clinical, histological, imaging, demographic, epidemiological data or combination of these. Papers that focus on the prediction of cancer development by means of conventional statistical methods (e.g. chi-square, Cox regression) were excluded as were papers that use techniques for tumor classification or identification of predictive factors. According to [3] and their survey based on ML applications in cancer prediction, we noted a rapid increase in papers that have been published in the last decade. Although it is impossible to achieve a complete coverage of the literature, we believe that a significant number of relevant papers were extracted and are presented in this review. As mentioned above, from the initial group of papers we selected a representative list that follows a well-organized structure. Specifically, we selected these studies that make use of recognizable ML techniques and integrated data from heterogeneous sources in order to predict the desirable outcome. We focused mainly on studies that have been published the last 5 years as an aim to present the most recent state of the art in the field and their advances in comparison to older publications. Table 1a, Table 1b, Table 1c depict some of the publications presented in this review. Cancer type, ML method, number of patients, type of data as well as the overall accuracy achieved by each proposed method are presented. Each sub-table corresponds to studies regarding a specific scenario (i.e. cancer susceptibility prediction, cancer recurrence prediction and cancer survival prediction). It should be noted that in articles that more than one ML techniques are applied for prediction, we decided to present here the most accurate predictive model.

A detailed analysis of more recent studies revealed that there is a growing trend in risk assessment as well as the prediction of recurrence of a cancer type regardless the ML technique used. Many research groups have tried to predict the possibility of redeveloping cancer after remission and appeared to improve the accuracy of predictions compared to alternative statistical techniques. Moreover, the vast majority of these publications used molecular and clinical data in order to make their predictions. The use of such measurable features as input data is a growing trend based on the advent of HTTs.

In the following, we are going to discuss one case for each of the objectives of predicting (i) susceptibility, (ii) recurrence and (iii) survival, all by means of ML techniques. Each sub-section summarizes the representative studies we have selected based on their predictive outcomes. We only selected those publications that have been accepted the last 5 years and make use of distinguishable ML methods. We provide the readers with the appropriate details of the most recent techniques used for the prediction and prognosis of most frequent cancer types.

### 4.1 Prediction of cancer susceptibility

We performed a Scopus and a PubMed advanced search which was limited to the last 5 years. Out of these results one of the publications employs ML techniques for the prediction of susceptibility in a cancer type [55]. The authors perform a genetic epidemiology study of bladder cancer susceptibility in terms of Learning Classifying Systems (LCSs). We decided to exclude this work from the present case study as it deals with genetic information and examines further genetic problems. Based on these limitations we continued our search to the specific biomedical databases. Most of these titles neither referred to the specified keywords that are mentioned in the relevant survey nor used ML techniques for their predictions. Among the most recent publications that resulted after our limited literature search regarding the cancer risk assessment prediction [19], [56], [57], [58], we selected a recent and very interesting study to present relevant to the breast cancer risk estimation by means of ANNs [19]. It is a different study among the others presented in this review article regarding the data type used. Although all of the publications selected make use of molecular, clinical or population-based data, this work encompasses mammographic findings and demographic characteristics to the model. Even though this work doesn't fit our general statement regarding our search criteria, we decided to include it in this case study because no other search result met our needs. We excluded this work from our general statement because no other search result met our needs. The major intense in developing decision-making tools that can discriminate among benign and malignant findings in breast cancer is commented by the authors. They also mention that when developing prediction models, risk stratification is of major interest. According to their knowledge, existing studies based on the use of computer models, have also utilized specific ML techniques, such as ANNs, in order to assess the risk of breast cancer patients. In their work, ANNs are employed in order to develop a prediction model that could classify malignant mammographic findings from benign. They built their model with a large number of hidden layers which generalizes better than networks with small number of hidden nodes. Regarding the collected data in this study, 48.774 mammographic findings as well as demographic risks factors and tumor characteristics were considered. All of the mammographic records were reviewed by radiologists and the reading information was obtained. This dataset was then fed as input to the ANN model. Its performance was estimated by means of ten-fold cross validation. Additionally, in order to prevent the case of overfitting the authors used the ES approach. This procedure, generally, controls the network error during training and stops it if overfitting occurs. The calculated AUC of their model was 0.965 following training and testing by means of ten-fold cross validation. The authors claimed that their model can accurately estimate the risk assessment of breast cancer patients by integrating a large data sample. They also declared that their model is unique among others if we consider that the most important factors they used to train the ANN model are the mammography findings with tumor registry outcomes. One very interesting characteristic in this study is the calculation of two main components of accuracy, namely discrimination and calibration. Discrimination is a metric that someone calculates in order to separate benign abnormalities from malignant ones, while calibration is a measurement used when a risk prediction model aims to stratify patients into high or low risk categories. The authors plotted (i) a ROC curve in order to evaluate the discriminative ability of their model and (ii) a calibration curve for comparing afterwards their model's calibration to the perfect calibration of predicting breast cancer risk. Apart from these findings, the authors also noted that the use of a mix of screening and diagnostic datasets cannot be reliably separated when feeding as input to the ANN. So, in order to overcome such limitations the authors should consider the purpose of preprocessing steps for transforming the raw data into appropriate formats for subsequent analysis.

### 4.2 Prediction of cancer recurrence

Based on our survey, we here present the most relevant and recent publications that proposed the use of ML techniques for cancer recurrence prediction. A work which studies the recurrence prediction of oral squamous cell carcinoma (OSCC) is proposed in [24]. They suggested a multiparametric Decision Support System in order to analyze the basis of OSCC evolvement after total remission of cancer patients. They exploited heterogeneous sources of data (clinical, imaging and genomic) in order to predict a possible relapse of OSCC and thus a subsequent recurrence. A total number of 86 patients were considered in this study, 13 of which have been identified with a relapse while the remaining was disease free. A specific feature selection procedure was followed with the employment of two feature selection algorithms, namely CFS [59] and wrapper algorithm [60]. As a result, any bias could be avoided when selecting the most informative features of their reference heterogeneous dataset. Then the selected important variables could be used as input vectors to specific classifiers. Before the employment of the feature selection techniques the total number of the clinical, imaging and genomic features was 65, 17 and 40 in each category. Subsequently, after the employment of the CFS algorithm the total number of clinical, imaging and genomic data used in each classifier was 8, 6 and 7, respectively. More specifically, among the clinical variables the most informative ones, for each classification algorithm, were the smoker, tumor thickness and p53 stain. Concerning the imaging and the genomic features, after the utilization of the CFS algorithm, the most important were the extra-tumor spreading, the number of lymph nodes and the SOD2, TCAM and OXCT2 genes.

The basic idea in this study is summarized in the discrimination of patients into those with a disease relapse and those without after the performance of five classification algorithms. The employed algorithms include the BNs, ANNs, SVMs, DTs and RF classifiers. After the performance of each ML method an evaluation technique, namely ten-fold cross-validation, was employed for evaluation purposes. Additionally, accuracy, sensitivity and specificity were also calculated for comparison reasons among the employed classification schemes. The analysis of ROC curve was considered by the authors for evaluation purposes as well. Their predictive results regarding the classification schemes employed were obtained based on the classification of data without performing feature selection and on the classification of data after employing a feature selection algorithm. Regarding their outputs the authors claimed that the BN classifier without applying any feature selection scheme performed better in the discrimination with directly input of the clinical and imaging features (78.6% and 82.8% accuracy, respectively). In a similar manner, genomic-based classification results revealed that the best performing classifier was the BN in conjunction with the CFS algorithm (91.7% accuracy). In the final stage of their study, the authors combined the more accurate individual predictors (i.e. BN and BN coupled with the CFS) in order to yield a consensus decision for discrimination between patients with and without an OSCC relapse. A comparison of this approach to other studies in the literature revealed that this proposal yields robust results than other methodologies. The proposed study illustrated in an explanatory way how the integration of heterogeneous sources of data, by means of ML classifiers, can produce accurate results regarding the prediction of cancer recurrence. Furthermore, the authors used more than one classification technique in order to obtain robust results. It is clear that when you estimate the performance of a classifier predictor among others, then you are able to find the most optimal tool. However, we should highlight an important aspect of this work regarding the small sample size. Only 86 patients were considered with their clinical, imaging and genomic features. Although their classification results were very promising, we should consider that a relatively small sample size compared to data dimensionality can lead to misclassification and biased predictors. Another interesting article published in the same year with [24] proposed an SVM-based model for the prediction of breast cancer recurrence, called BCRSVM [47]. The authors support the idea that the classification of cancer patients into high-risk or low-risk groups allows experts to adjust a better treatment and follow-up planning. Their study is based on the development of a predictive model regarding the breast cancer recurrence within five years after surgery. SVM, ANN as well as Cox-proportional hazard regression were employed for producing the models and find the optimal one. The authors claimed that after comparing the three models based on their resulted accuracies, they found that the BCRSVM model outperformed the other two. From the initial set of 193 available variables in their dataset, only 14 features were selected based on their clinical knowledge. These data refer to clinical, epidemiological and pathological variables of 733 patients considered out of 1.541. In the final stage of the feature selection, Kaplan–Meier analysis and Cox regression were applied which resulted in 7 variables as most informative. These features were then entered as input to the SVM and ANN classifiers as well as to the Cox regression statistical model. In order to evaluate the performance of the models, the authors employed the hold-out method, which splits the data sample into two sub-sets, namely training and testing set. As in most studies in the literature, accuracy, sensitivity and specificity were calculated for a reliable estimation of the models. Based on these metrics, the authors claimed that BCRSVM outperformed the ANN and Cox regression models with accuracy 84.6%, 81.4% and 72.6%, respectively. Comparison among the performance of other previously established recurrence prediction models revealed that BCRSVM has superior performance. It should be noted that this study estimated also the importance of prognostic factors by means of normalized mutual information index (NMI) [61]. Based on these calculations for each of the three predictive models, they suggest that the most significant factor regarding the prediction of breast cancer recurrence was the local invasion of tumor. However, if someone reviews this work would certainly mention some major limitations. As the authors noted, the exclusion of a large number of patients (n = 808) due to the lack of clinical data in the research registry, influenced the performance of their models. Furthermore, the fact that the authors used only their clinical knowledge to select 14 out of 193 variables may have resulted in significant bias, thus giving no robust results. Apart from this limitation, the authors could also improve the performance of their proposed model, namely BCRSVM, by validating it with external datasets from other sources. Among the initial list of publications resulted from our literature survey, we noticed a growing trend the last years regarding the prediction of cancer disease by means of SSL learning. So, we believed it would be of interest to present the most recent study that makes use of this type of ML techniques for the analysis of breast cancer recurrence [48]. The proposed algorithm is based on the use of SSL for the construction of a graph model while it integrates gene expression data with gene network information in order to predict cancer recurrence. Based on biological knowledge, the authors selected gene pairs that indicate strong biological interactions. The sub-gene network identified by the proposed method is constituted of the BRCA1, CCND1, STAT1 and CCNB1 genes. Their methodology is divided in three sections including: (i) the determination of gene pairs for building the graph model with only labeled samples, (ii) the development of sample graphs based on informative genes and (iii) the regularization of the graph resulting in finding the labels of unlabeled samples. The dataset used through this study consists of gene expression profiles found in the GEO repository [62] as well as of PPIs derived from the I2D database [63]. Specifically, five gene expression datasets were downloaded from GEO including 125, 145, 181, 249 and 111 labeled samples. These samples were classified into three groups: (i) recurrence, (ii) non-recurrence and (iii) unlabeled samples and referred to cancer types like breast and colon cancer. Additionally, they downloaded from the I2D database a sample of human PPIs composed of 194.988 known, experimental and predicted interactions. After removing the duplicated PPIs and the interactions that do not contain proteins mapped to a gene they resulted in an amount of 108,544 interactions. Based on the results of this study, the authors showed that the gene networks derived from the SSL learning method include many important genes related to cancer recurrence. They also claimed that their approach outperforms other existing methods in the case of breast cancer recurrence prediction. The estimated performance of the proposed method compared to other known methods that make use of PPIs for the identification of informative genes showed an accuracy of 80.7% and 76.7% in the breast and colon cancer samples, respectively. Ten-fold cross validation was used for estimating the experimental results. Although this type of ML methods differs considerably from these of supervised and unsupervised learning on the algorithms that they employ, it is clear that it provides more advantages relevant to the collection of datasets and their sizes. Unlabeled data are cheap and can be easier extracted. On the contrary, labeled samples may require experts and special devices in order to be collected. This study reveals that SSL can be an alternative to supervised approaches which usually suffers from small labeled samples.

### 4.3 Prediction of cancer survival

In [26] a predictive model is developed for the evaluation of survival in women that have been diagnosed with breast cancer, while they addressed the importance of robustness under the model's parameter variation. They compared three classification models namely SVM, ANN and SSL based on the SEER cancer database [64]. The dataset is composed of 162,500 records with 16 key features. A class variable was also considered, namely survivability, referring to patients that had not survived and those that had survived. Among the most informative features are (i) the tumor size, (ii) the number of nodes and (iii) the age at diagnosis. By comparing the best performance of each of the three models they found that the calculated accuracy for ANN, SVM and SSL was 65%, 51% and 71% respectively. Five-fold cross validation was used for evaluating the performance of the predictive models. Concerning those findings the authors proposed the SSL model as a good candidate for survival analysis by the clinical experts. We should note that no preprocessing steps were mentioned by the authors regarding the collection of the most informative features. They proceeded with the entire SEER datasets and the box-whisper-plot was used for estimating the performance variation across 25 combinations of model parameters. A small box area of a specific model indicates more robustness and stability under parameter combination. The small boxes of the SSL model revealed its better accuracy than the other models. A relevant study was published the next year which attempts to assess the survival prediction of non-small cell lung cancer (NSCLC) patients through the use of ANNs [50]. Their dataset consists of NSCLC patients' gene expression raw data and clinical data obtained from the NCI caArray database [65]. After the preprocessing steps in their approach, the authors selected the most informative survival-associated gene signatures; LCK and ERBB2 genes, which were then used for training the ANN network. Four clinical variables, namely sex, age, T_stage and N_stage were also considered as input variables in the ANN model. They also performed several types of ANN architectures in order to find the optimal one for the prediction of cancer survival. An overall accuracy of 83% was provided regarding the predictive performance of the classification scheme. Furthermore, their results revealed that all patients were classified in different groups regarding their treatment protocol while 50% of them had not survived. The evaluation of the model outcomes was done based on the Kaplan–Meier survival analysis. They estimated the survival of patients for the training set, the test set and the validation set with p-value < 0.00001, while they showed that the patients in the high-risk group exhibited a lower median overall survival in comparison to low-risk patients. Compared to other studies in the literature relevant to NSCLC survival prediction, this work provided more stable results. However, existing limitations of the current article are related to the fact that the impact of other variables related to death (such as blood clots) is not considered, which may have led to misclassification results. Furthermore, the authors claim that their model could not be applied to other cancer types except NSCLC. This assumption is considered as a major limitation in studies that the predictive models may not generalize to different cancer types.

## 5 Discussion

In the present review, the most recent works relevant to cancer prediction/prognosis by means of ML techniques are presented. After a brief description of the ML branch and the concepts of the data preprocessing methods, the feature selection techniques and the classification algorithms being used, we outlined three specific case studies regarding the prediction of cancer susceptibility, cancer recurrence and cancer survival based on popular ML tools. Obviously, there is a large amount of ML studies published in the last decade that provide accurate results concerning the specific predictive cancer outcomes. However, the identification of potential drawbacks including the experimental design, the collection of appropriate data samples and the validation of the classified results, is critical for the extraction of clinical decisions.

Moreover, it should be mentioned that in spite of the claims that these ML classification techniques can result in adequate and effective decision making, very few have actually penetrated the clinical practice. Recent advances in omics technologies paved the way to further improve our understanding of a variety of diseases; however more accurate validation results are needed before gene expression signatures can be useful in the clinics.

A growing trend was noted in the studies published the last 2 years that applied semi-supervised ML techniques for modeling cancer survival. This type of algorithms employs labeled and unlabeled data for their predictions while it has been proven that they improved the estimated performance compared to existing supervised techniques [26]. SSL can be though as a great alternative to the other two types of ML methods (i.e. supervised learning and unsupervised learning) that use, in general, only a few labeled samples.

One of the most common limitations noted in the studies surveyed in this review is the small amount of data samples. A basic requirement when using classification schemes for modeling a disease is the size of the training datasets that needs to be sufficiently large. A relatively large dataset allows the sufficient partitioning into training and testing sets, thus leading to reasonable validation of the estimators. A small sized training sample, compared to data dimensionality, can result in misclassifications while the estimators may produce unstable and biased models. It is obvious that a richer set of patients used for their survival prediction can enhance the generalizability of the predictive model.

Except the data size, the dataset quality as well as the careful feature selection schemes are of great importance for effective ML and subsequently for accurate cancer predictions. Choosing the most informative feature subset for training a model, by means of feature selection methods, could result in robust models. Additionally, feature sets that consist of histological or pathological assessments are characterized by reproducible values. Due to the lack of static entities when dealing with clinical variables it is important for a ML technique to be adjusted to different feature sets over time.

It should be noted that almost all of the works presented here, performed validation tests for estimating the performance of their learning algorithms. They employed well-known evaluation techniques that split the initial datasets into subsets. As mentioned above, in order to obtain accurate results for their predictive models, the authors should select large and independent features that could result in better validation. Internal and external validation was performed in these studies that would enable the extraction of more accurate and reliable predictions while it would minimize any bias [47].

A key point to several studies, regarding their promising results, was the fact that several ML techniques were employed as an aim to find the most optimal one [34]. Apart from this, the combination of multiple data types that would be fed as input to the models is also a trend. Looking back to the previous decade, only molecular and clinical information was exploited for making predictions of cancer outcomes. With the rapid development of HTTs, including genomic, proteomic and imaging technologies, new types of input parameters have been collected. We found that almost all the predictions was made by integrating either genomic, clinical, histological, imaging, demographic, epidemiological data and proteomic data or different combinations of these types [24], [26], [48], [50], [53].

Additionally, there has been considerable activity regarding the integration of different types of data in the field of breast cancer [66], [67]. In the DREAM project [68], several attempts to combine clinical treatment scores with signatures based on immunohistochemistry [69] as well as expression-based signatures such as PAM50 [70] and Oncotype DX [71] reveal the extensive work done for improving treatment based on the incorporation of different features.

Among the most common applied ML algorithms relevant to the prediction outcomes of cancer patients, we found that SVM and ANN classifiers were widely used. As mentioned to our introductory section, ANNs have been used extensively for nearly 30 years [30]. In addition, SVMs constitute a more recent approach in the cancer prediction/prognosis and have been used widely due to its accurate predictive performance. However, the choice of the most appropriate algorithm depends on many parameters including the types of data collected, the size of the data samples, the time limitations as well as the type of prediction outcomes.

Concerning the future of cancer modeling new methods should be studied for overcoming the limitations discussed above. A better statistical analysis of the heterogeneous datasets used would provide more accurate results and would give reasoning to disease outcomes. Further research is required based on the construction of more public databases that would collect valid cancer dataset of all patients that have been diagnosed with the disease. Their exploitation by the researchers would facilitate their modeling studies resulting in more valid results and integrated clinical decision making.

## 6 Conclusions

In this review, we discussed the concepts of ML while we outlined their application in cancer prediction/prognosis. Most of the studies that have been proposed the last years and focus on the development of predictive models using supervised ML methods and classification algorithms aiming to predict valid disease outcomes. Based on the analysis of their results, it is evident that the integration of multidimensional heterogeneous data, combined with the application of different techniques for feature selection and classification can provide promising tools for inference in the cancer domain.

## Figures and Tables

**Table 1a:** Publications relevant to ML methods used for cancer susceptibility prediction.

Publication	Method	Cancer type	No of patients	Type of data	Accuracy	Validation method	Important features
Ayer T et al. [19]	ANN	Breast cancer	62,219	Mammographic, demographic	AUC = 0.965	10-fold cross validation	Age, mammography findings
Waddell M et al. [44]	SVM	Multiple myeloma	80	SNPs	71%	Leave-one-out cross validation	snp739514, snp521522, snp994532
Listgarten J et al. [45]	SVM	Breast cancer	174	SNPs	69%	20-fold cross validation	snpCY11B2 (+) 4536 T/C snpCYP1B1 (+) 4328 C/G
Stajadinovic et al. [46]	BN	Colon carcinomatosis	53	Clinical, pathologic	AUC = 0.71	Cross-validation	Primary tumor histology, nodal staging, extent of peritoneal cancer

**Table 1b:** Publications relevant to ML methods used for cancer recurrence prediction.

Publication	ML method	Cancer type	No of patients	Type of data	Accuracy	Validation method	Important features
Exarchos K et al. [24]	BN	Oral cancer	86	Clinical, imaging tissue genomic, blood genomic	100%	10-fold cross validation	Smoker, p53 stain, extra-tumor spreading, TCAM, SOD2
Kim W et al. [47]	SVM	Breast cancer	679	Clinical, pathologic, epidemiologic	89%	Hold-out	Local invasion of tumor
Park C et al. [48]	Graph-based SSL algorithm	Colon cancer, breast cancer	437 374	Gene expression, PPIs	76.7% 80.7%	10-fold cross validation	BRCA1, CCND1, STAT1, CCNB1
Tseng C-J et al. [49]	SVM	Cervical cancer	168	Clinical, pathologic	68%	Hold-out	pathologic_S, pathologic_T, cell type RT target summary
Eshlaghy A et al. [34]	SVM	Breast cancer	547	Clinical, population	95%	10-fold cross validation	Age at diagnosis, age at menarche

**Table 1c:** Publications relevant to ML methods used for cancer survival prediction.

Publication	ML method	Cancer type	No of patients	Type of data	Accuracy	Validation method	Important features
Chen Y-C et al. [50]	ANN	Lung cancer	440	Clinical, gene expression	83.5%	Cross validation	Sex, age, T_stage, N_stage LCK and ERBB2 genes
Park K et al. [26]	Graph-based SSL algorithm	Breast cancer	162,500	SEER	71%	5-fold cross validation	Tumor size, age at diagnosis, number of nodes
Chang S-W et al. [32]	SVM	Oral cancer	31	Clinical, genomic	75%	Cross validation	Drink, invasion, p63 gene
Xu X et al. [51]	SVM	Breast cancer	295	Genomic	97%	Leave-one-out cross validation	50-gene signature
Gevaert O et al. [52]	BN	Breast cancer	97	Clinical, microarray	AUC = 0.851	Hold-Out	Age, angioinvasion, grade MMP9, HRASLA and RAB27B genes
Rosado P et al. [53]	SVM	Oral cancer	69	Clinical, molecular	98%	Cross validation	TNM_stage, number of recurrences
Delen D et al. [54]	DT	Breast cancer	200,000	SEER	93%	Cross validation	Age at diagnosis, tumor size, number of nodes, histology
Kim J et al. [36]	SSL Co-training algorithm	Breast cancer	162,500	SEER	76%	5-fold cross validation	Age at diagnosis, tumor size, number of nodes, extension of tumor
